# Knowledge of vitamin D and perceptions and attitudes toward sunlight among Chinese middle-aged and elderly women: a population survey in Hong Kong

**DOI:** 10.1186/1471-2458-6-226

**Published:** 2006-09-07

**Authors:** Annie WC Kung, Ka-Kui Lee

**Affiliations:** 1 Department of Medicine, The University of Hong Kong, Hong Kong, China

## Abstract

**Background:**

Physical and biological risk factors for vitamin D inadequacy are known; however, cultural- and population-specific behaviours and attitudes that influence these risk factors, particularly among Asian people, are less well documented. To understand more about prevailing attitudes and behaviour toward sunlight and knowledge of vitamin D among a population at greater risk of impaired vitamin D status, poor bone health and osteoporosis, we conducted a telephone interview survey of 547 middle-aged and elderly Chinese women living in Hong Kong.

**Methods:**

All telephone interviews were conducted using the Computer Assisted Telephone Technique and target respondents were selected by random sampling. Interviews were conducted in Cantonese and eighteen main questions were asked pertaining to personal characteristics, perceptions, attitudes and behaviour toward sunlight, and knowledge about vitamin D.

**Results:**

The survey results showed that 62.3% (n = 341) did not like going in the sun and 66.7% of respondents spent an average of 6–10 hours indoors, between 6:30 am and 7:00 pm, during weekdays. However, 58% of people thought that they had enough exposure to sunlight. The majority had heard of vitamin D, but knowledge about the role and sources of vitamin D was low. Among those who knew that sunlight was a source of vitamin D, the majority spent less than 1 h in the sun in the past week (76.4% vs 23.6%, < 1 h in the sun in the past week vs > 1 h in the sun in the past week, chi-square p < 0.05). There were significantly more users of sunscreen products (75.5% vs 53.0%, p < 0.0001, sunscreen users vs non-users) and parasols (68.4% vs 43.7%, p < 0.0001, parasol users vs non-users) among respondents who knew that vitamin D was good for bone health and that sunlight was a source of vitamin D. Age, occupation, subjects who liked going in the sun were factors associated with awareness of vitamin D but age was the only predictive factor for giving correct answers to the actions and sources of vitamin D.

**Conclusion:**

The survey revealed considerable ignorance and confusion about the role of sunlight in vitamin D production, and the function and sources of vitamin D. Attitudes and behaviour toward sunlight were largely negative and many took measures to avoid sunlight, particularly among younger (middle-aged) women who had good awareness of vitamin D.

## Background

Vitamin D plays a central role in bone metabolism and maintaining bone health, and is important for muscle functioning [1]. The long-term effects of vitamin D inadequacy on calcium homeostasis have been associated with the development of chronic bone disorders, such as osteoporosis [1,2]. Despite this, there is a worldwide problem of vitamin D inadequacy and deficiency that is largely being unheeded and under-treated. This problem is not isolated, but affects developed as well as developing countries, subtropical and temperate regions, and populations of all ages [3-8]. Vitamin D inadequacy and deficiency may pose a bigger threat in subtropical Asian countries, as the assessment of vitamin D status and vitamin D education are largely overlooked, perhaps on the assumption that vitamin D insufficiency is unlikely to occur in regions with plentiful sunshine.

The main sources of vitamin D are UV-B radiation from the sun, dietary intake, and vitamin D dietary supplements. However, few foods are naturally rich in vitamin D – these include oily fish, cod-liver oil and egg yolks. Though the physical and biological factors contributing to impaired vitamin D status are known [1,2], the influence of cultural-specific practices and behaviour on these risk factors, and the motivation for these behaviours, are less well documented, particularly in Asians.

The reported incidence of hip fracture in Hong Kong, a city situated on the southern coast of China at a latitude of 22.5 degrees north, has more than doubled in the last three decades [9]. Rapid industrialization in Hong Kong and southern China has considerably worsened air pollution, which potentially reduces the penetration of UV-B radiation from the sun [10]. Furthermore, the typical Chinese diet is low in calcium, there are few foods fortified with vitamin D available in Hong Kong and, as in other developed nations, the population is shifting towards an increasingly sedentary lifestyle spent predominantly indoors.

To assess the knowledge of people in Hong Kong about vitamin D, and attitudes and behaviour toward sunlight exposure, we conducted a population survey of middle-aged and elderly women who are likely to be at increased risk of vitamin D inadequacy and osteoporosis.

## Methods

All telephone interviews were conducted in Hong Kong using the Computer Assisted Telephone Interview (CATI) technique. Calls were made by experienced telephone interviewers trained by the Social Sciences Research Centre of The University of Hong Kong. Telephone calls were made from 4:00 pm to 10:30 pm in November 2005. Evening time was chosen for interview to increase the success rate, as it was expected that the majority of younger subjects would be workers. The target respondents – Chinese female household members aged 50 years or older living in Hong Kong – were selected for telephone interview by random sampling to produce a carefully constructed representation of the population. In each successfully contacted residential unit, only one person aged 50 years or older was selected for interview by the modified "Last Birthday" method: the household member with the most recent last birthday and was present at home at the time of interview was selected. Interviews were conducted in Cantonese, the local southern Chinese dialect. Consent was signed by individuals willing to participate in the study. The study was approved by the Ethics Committee of the University of Hong Kong and was conducted according to the Declaration of Helsinki.

### Survey questions

A pilot survey was performed and validated against face-to-face interviews, and the wording of questions was refined and retested prior to the actual survey. The survey comprised eighteen main questions, pertaining to personal characteristics, perceptions, attitudes and behaviour toward sunlight, and knowledge about vitamin D, with multiple-choice answers (Table 1). Respondents were permitted to abstain from answering and these answers were captured as missing responses in the data analysis. The age grouping and job classification of respondents were similar to that utilized in the census survey of the Hong Kong SAR Government.

### Data analysis

The results were analysed using SPSS version 10.0. The duration of outdoor activities and exposure to sunlight were determined and correlated to job classification. Correlations and comparisons were made using the Kruskal-Wallis test and chi-square test. The awareness and understanding of the role of vitamin D, as assessed by valid responses to questions on the source and action of vitamin D, were compared and correlated to age, job classification, duration of outdoor activities and duration of sunlight exposure. Logistic regression analysis was performed to identify factors that determined the level of awareness of vitamin D.

## Results

A sample of 20,602 telephone numbers was drawn and contacted. In total, 547 of 1,014 valid respondents were successfully interviewed using CATI, resulting in an overall response rate of 53.94%. The contact rate was 45.52%, based on 9,378 answered calls of 20,602 attempted calls. Personal characteristics of the surveyed population are given in Table 2.

### Attitudes and behaviour toward sunlight exposure

Of those surveyed, 62.3% (n = 341) responded that they did not like going in the sun. When the response was stratified by age group, it was found to be similar for all age groups apart from those ≥ 85 years, who were approximately twice as likely as other age groups to enjoy going in the sun (p < 0.05). Almost half (n = 243, 44.4%) used a parasol to shade themselves from the sun, but the majority (n = 444, 81.2%) did not use sunscreen products with sun-protection factor (SPF) ≥ 15. The most frequent use of sunscreen (30.6%) was among the youngest age group of 50–54 years. Notably, those who did not like going in the sun were significantly more likely to use a parasol than those who did like being in the sun (56.6% vs 24.3%, chi-square p < 0.0001).

On workdays (Monday through Saturday), 65.8% of respondents spent an average of 6–10 h indoors each day during the daylight hours of 6:30 am to 7:00 pm. Similarly, on Sundays (a public holiday in Hong Kong), 57.8% of respondents spent an average of 6–10 hours indoors from 6:30 am to 7:00 pm. The answers to the 4 questions on time spent indoors and outdoors on weekdays and weekends correlated significantly with each other, with the time spent indoors on weekdays and weekends being positively correlated (all p < 0.001), and the time spent indoors and outdoors negatively correlated (all p < 0.001), suggesting that the respondents were consistent in their replies during the interview.

When the respondents were asked how much time they had spent in the sun in the past week, 14.8% of subjects replied less than 15 min, 8.2% replied between 15 to 30 min, 5.7% replied between 30 to 60 min and 11.0% replied between 1 to 2 hours. Only 56.9% of respondents had spent 2 hours or more under the sun in the past week. Just over half of those surveyed (n = 303, 55.4%) said they liked doing outdoor activities, but 30.2% of people took part in outdoor activities less than once a month and 10.1% (n = 55) never undertook outdoor activities.

Nevertheless, 58% (n = 317) of the people surveyed thought they had enough exposure to sunlight. This self-evaluation was significantly correlated with the duration of sunlight exposure (p < 0.001) and duration of outdoor activities (p < 0.005).

### Awareness and knowledge of the role of vitamin D

**Figure 1:**
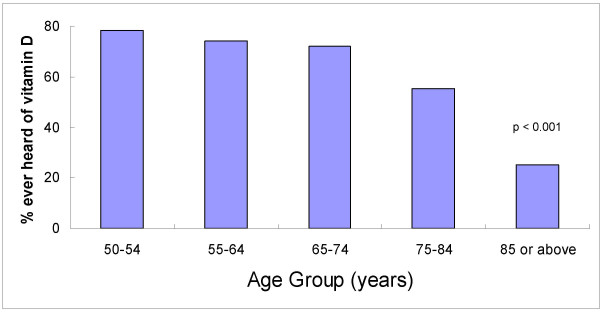
Age stratification of response to the question "Have you heard of vitamin D?".

When asked if they had heard of vitamin D, 397 respondents (72.6%) replied yes. The rate of positive response to this question differed significantly between age groups and occupation classification: 78.3% of those aged 50–54 years had heard of vitamin D compared with only 25.0% of those aged ≥ 85 years (p < 0.001 vs other age groups; Figure 1).

Although the majority of surveyed subjects had heard of vitamin D, only 176 of 547 cases (32.2%) gave valid responses to the question, "What does vitamin D do?", and the response was mixed. The three most frequent responses were bone strengthening (n = 97, 17.7%), enhance calcium absorption (n = 66, 12.0%) and build immunity (n = 21, 3.8%).

**Figure 2:**
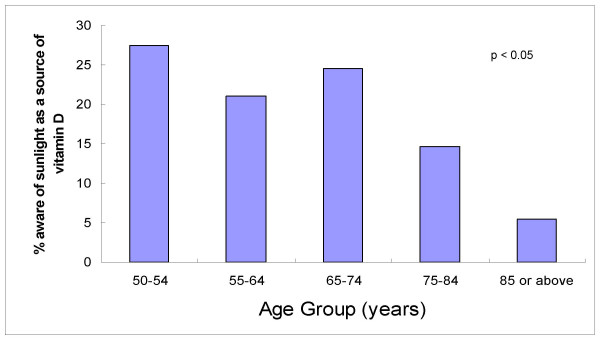
Age stratification of response to the question "Did you know that sun exposure can give you vitamin D?".

There was a similarly low and mixed response rate – 210 of 547 cases (38.4%) giving valid responses – when respondents were asked the sources of vitamin D. The three most frequent responses were sunlight (n = 127, 23.2%), vegetables (n = 78, 14.3%) and dairy products (n = 49, 9.0%). Only 3 people (0.5%) thought cod-liver oil or eggs were sources of vitamin D and 5 subjects (0.9%) gave a response of vitamin supplements. There was a significant relationship between valid response for sunlight as a source of vitamin D with younger age (p < 0.05; Figure 2), but not with occupation.

When subjects were asked the question "Have you ever heard that vitamin D is good for bone health?". 56.7% (310/547) of the surveyed population gave a positive response, but 42.4% did not think that vitamin D was good for bone health and 5 subjects (0.9%) did not know either way. When respondents were asked how they had learnt this, 55.6% said that they had acquired this information from the media compared with 8.0% who had learnt from their doctors, 6% from friends and family members, and 4.2% from books.

Lastly, when the subjects were asked the question, "Do you know that sun exposure can give you vitamin D?", 52.6% (n = 288) replied yes. However, only 12.4% (n = 68) knew that it took less than 15 minutes of daily sun exposure to acquire sufficient vitamin D. Moreover, 70.1% of subjects abstained from answering this question. Of the 29.9% of subjects who were able to reply, 26.1% said they had acquired this knowledge from the media, 4.4% from their doctors, 9.1% from school, 5.9% from friends and family members, and 3.7% from books.

We dichotomized the response for time spent in the sun in the past week to more or less than 1 hour among those who gave a valid response for sunlight being a source of vitamin D. This gave the surprising result that subjects who spent less than 1 h in the sun in the past week were more likely to know that sunlight was a source of vitamin D (76.4% vs 23.6%, < 1 h in the sun in the past week vs > 1 h in the sun in the past week, chi-square p < 0.05). Similarly, there was no relationship between those who gave a valid response for sunlight being a source of vitamin D and those who thought they had enough exposure to sunlight (chi-square p = not significant). Furthermore, there were significantly more users of sunscreen products (75.5% vs 53.0%, p < 0.0001, sunscreen users vs non-users) and parasols (68.4% vs 43.7%, p < 0.0001, parasol users vs non-users) among those respondents who said they knew vitamin D was good for bone health and that sunlight was a source of vitamin D.

The logistic regression analysis model was used to determine the factors predicting the level of awareness and knowledge of vitamin D. Age, occupation, subjects who liked going in the sun and subjects who spent more than 2 hours in the sun in the past week were factors associated with a positive response to the question, "Have you heard of vitamin D". Age was the only predictive factor for giving correct answers to the two questions on the action of vitamin D and the sources of vitamin D.

## Discussion

This survey provides insight into the current awareness and understanding of the role of vitamin D and attitudes toward sunlight exposure among a female Chinese population. Maintaining good bone health is important in this population, particularly with regard to preventing or reducing the risk of osteoporosis. As in other parts of the world, vitamin D inadequacy is prevalent among the general Hong Kong population. We previously showed in a cross-sectional study of ambulatory, community-dwelling Hong Kong adults (n = 382; mean age, 69 years [range 50–91]) that 22.5% of study subjects had serum 25-hydroxyvitamin D [25(OH)D] levels less than 20 ng/mL, while 62.8% had levels less than 30 ng/mL [11]. In this population, 25(OH)D levels of 30 ng/mL or lower were inversely correlated with parathyroid hormone (PTH) levels [11]. Subjects with secondary hyperparathyroidism had significantly higher risks of falls and osteoporotic fracture at the hip and spine [11].

### Attitudes toward sunlight exposure and awareness of vitamin D

Although older women (75–84 years and ≥ 85 years) were less aware of vitamin D than women in the younger age groups, they were more likely to go in the sun than their younger counterparts. These results are compatible with findings from an earlier Hong Kong study, which showed adults aged 50–60 years to have the lowest mean 25(OH)D levels [11].

Middle-aged women in the survey were also the greatest users of sunscreen products and parasols, which tends to suggest a greater, inherent aversion to sunlight than elderly women. SPF > 15 was chosen because sunscreen products with SPF < 15 are not commercially available in Hong Kong. It has been documented that products with SPF 8 are already sufficient to reduce the skin's ability to produce vitamin D by 95% [12].

The reasons for sunlight avoidance were not investigated in this survey, but it can be speculated that the current cultural trend (prevalent among many Asian female populations), and even pressure, to have paler skin is a contributing factor for reducing sunlight exposure among the younger age groups in the survey. It can also be speculated that awareness of the harmful effects of sunlight (for example, skin ageing, skin cancer) has a greater impact and influence on behaviour among middle-aged women than any knowledge about the benefits of sunlight.

In the current study, only the total length of time spent in the sun from 6.30 am to 7.00 pm was surveyed and no further documentation of the usual time of day spent in the sun by respondents was recorded, which somewhat limits the study analysis. As previous data have suggested that early morning, late afternoon and evening exposure to sunlight may not result in any vitamin D production even in the tropics [13], it is likely that the percentage of respondents with adequate sunlight exposure might be even lower than that estimated from the current survey results.

Finally, in addition to less inclination, the survey found that the lifestyles of middle-aged Hong Kong women in this population reduced their opportunities for sunlight exposure. Lifestyle factors contributing to reduced sunlight exposure were spending many daylight hours indoors (eg, due to work) and lack of regular participation in outdoor activities.

### Ignorance about the role and action of vitamin D

Despite greater awareness of vitamin D among the younger women in the survey, ignorance and confusion about the role and sources of vitamin D were common in all age groups and across all occupations. This was revealed by the low response rates and mixed responses to the questions: "What does vitamin D do?"; "What are the sources of vitamin D?"; and "How much time do you need to spend in the sun to get enough vitamin D?"

The finding that the media appear to be the main source of information on vitamin D for this population is also of some concern, and may have further contributed to current misconceptions about vitamin D and sunlight exposure. Nevertheless, the results suggest that the mass media has an important role to play in public health education and a potential role in modifying behaviour of the population.

### Conflict between knowledge and behaviour

The survey results also highlighted a general trend for gaps or conflict between knowledge and behaviour. Specifically, those who knew about the benefits of sunlight and vitamin D for bone health, and were aware of sunlight as a source of vitamin D, comprised the majority of subjects most likely to avoid sunlight by using sunscreen and parasols, and staying indoors.

The motivations for this contradictory behaviour are intriguing and merit further investigation. Among this group, behaviour is apparently not being influenced by lack of knowledge about the benefits of sunlight and vitamin D – thus, to change behaviour and attitudes toward sunlight in this group, it would be important to learn why these women continue to avoid sunlight despite knowing of its benefits.

A limitation of the study was the response rate of the survey, which was modest at 54%. The study also did not address the reasons for sunlight avoidance in the surveyed population. Although the sample size in the older age groups was small, the overall age structure was similar to that of the general population in Hong Kong, as obtained from the census survey. The percentage of subjects in each job classification was also similar to that of the general population, with about 60% of respondents having occupations of indoor work. Further information on education and salary was not obtained as these data were closely correlated to occupation, according to the census survey conducted by the Hong Kong Government.

## Conclusion

This survey highlights some current trends and conflicts in attitudes and behaviour toward sunlight exposure, and knowledge of vitamin D, that may adversely affect vitamin D status in middle-aged and elderly Hong Kong women – a population at greater risk of poor bone health and vitamin D inadequacy. Overall, there is considerable underlying ignorance and confusion about the role of sunlight in vitamin D production, the benefits of sunlight, and the function and sources of vitamin D. Attitudes toward sunlight exposure are largely negative – many of the surveyed population disliked being in the sun and took measures to avoid sunlight.

Confirmation and further examination of these results, perhaps in a larger population study, may teach us more about the attitudes and beliefs of Hong Kong people toward sunlight and vitamin D. Moreover, greater understanding of underlying ignorance, cultural behaviours and practices that contribute to vitamin D inadequacy presents a public health opportunity to re-address misconceptions, and to develop education strategies that are targeted and specific for susceptible populations. Ultimately, improving knowledge and public health education to tackle modifiable preconceptions and behaviour may be an effective first step toward increasing individual responsibility for preventing vitamin D inadequacy and osteoporosis. The incidence of osteoporotic hip fracture in Hong Kong has increased dramatically in the last 30 years – if the risk of osteoporotic fracture is to be minimized in the future, particularly among high-risk groups such as middle-aged adults, the problem of vitamin D inadequacy needs to be addressed and prevention strategies implemented.

## Competing interests

The author(s) declare that they have no competing interests.

## Authors' contributions

AWCK and KKL conceived and designed the survey. AWCK analysed the survey results and was involved in writing, reading and editing the manuscript.

## Pre-publication history

The pre-publication history for this paper can be accessed here:



## Figures and Tables

**Table 1:** Survey questions

1. What is your age group?
2. What is your occupation?
3. Do you work mainly indoors or outdoors?
4. Do you like going in the sun?
5. Do you often use a parasol to shade from the sun?
6. Do you use sunscreen products containing SPF ≥ 15?
6.1 How often do you use these products?
7. On an average weekday, from 6:30 am to 7:00 pm, how many hours do you spend indoors?
8. On an average Sunday, from 6:30 am to 7:00 pm, how many hours do you spend indoors?
9. Do you like outdoor activities?
10. How often do you do outdoor activities?
11. On an average weekday, from 6:30 am to 7:00 pm, how many hours do you spend outdoors?
12. On an average Sunday, from 6:30 am to 7:00 pm, how many hours do you spend outdoors?
13. In the past week, how much time have you spent in the sun?
14. Do you think you have enough exposure to sunlight?
15. Have you ever heard of vitamin D?
15.1 What does vitamin D do?
16. What are the sources of vitamin D?
17. Do you know that vitamin D is good for bone health?
17.1 From where did you learn this?
18. Do you know that sunlight can give you vitamin D?
18.1 How much time do you need to spend in the sun to get enough vitamin D?
18.2 From where did you learn about this?

**Table 2:** Personal characteristics of female survey subjects (n = 547)

**Characteristics**	**n (%)**
Age	
50–54 years	180 (32.9%)
55–64 years	192 (35.1%)
65–74 years	115 (21.0%)
75–84 years	49 (9.0%)
≥85 years	8 (1.5%)
No data (refused to answer)	3 (0.5%)

Occupation	
Managers/administrator/professional	14 (2.6%)
Clerical	54 (9.9%)
Technical/manual	44 (8.0%)
Home-maker	250 (45.7%)
Retired	165 (30.2%)
Unemployed	16 (2.9%)
No data (refused to answer)	4 (0.7%)
